# Receive coil quality assurance procedure and automated analysis for ViewRay MRIdian MR‐Linac

**DOI:** 10.1002/acm2.14275

**Published:** 2024-01-17

**Authors:** Evangelia Kaza, Christopher L. Williams

**Affiliations:** ^1^ Radiation Oncology Brigham and Women's Hospital Dana‐Farber Cancer Institute Harvard Medical School Boston Massachusetts USA

**Keywords:** MRIdian, MR‐Linac, quality assurance, SNR, uniformity

## Abstract

**Purpose:**

Regular receiving coil quality assurance (QA) is required to ensure image quality of an MRIdian Linac system. The manufacturer provides a spherical phantom and positioning tube for single‐slice signal‐to‐noise ratio (SNR) and uniformity assessments. We aimed to improve imaging setup and coverage and eliminate inter‐scan variability by employing multi‐slice imaging of a stable phantom. Additionally, we strived to expedite analysis by developing objective, automated analysis software.

**Methods:**

A 5300 mL cylindrical plastic bottle placed in plastic bins was scanned at isocenter using a spin‐echo sequence with NEMA‐recommended parameters and 18 axial slices, avoiding phantom repositioning. Acquisition was repeated with and without prescan normalization filtering and by saving uncombined element images. Obtained data were analyzed using custom open‐source MATLAB code. Signal and noise images were automatically assigned, and ROIs for SNR and uniformity calculations were defined using image thresholding. SNR and uniformity pass/fail decisions were made using baseline comparisons.

**Results:**

The proposed method was successfully implemented as monthly coil QA for 3.5 years. Setup and scanning took 41 min on average for a coil set. Automated image analysis was completed in a few minutes. Signal intensity peaked around +90 or ‐90 mm for Torso or Head/Neck coil unfiltered images. Noise peaked and minimized SNR inside ±30 mm from isocenter, while maximizing it around ±130 mm. Prescan normalization smoothed signal response, reduced SNR and increased uniformity. Individual coil element image analysis identified their position, signal or noise response and SNR. SNR and uniformity pass/fail thresholds were set for already tested and new coils. Conspicuous and subtle Torso coil malfunctions were detected considering baseline deviations of combined and individual element results.

**Conclusions:**

Our QA method eliminated observer bias and provided insights into coil function, image filtering performance and coil element location. It provided SNR and uniformity thresholds and identified faulty coil elements.

## 1 INTRODUCTION

The ViewRay MRIdian Linac system is a hybrid medical device combining a magnetic resonance imaging system (MRIS) with a linear accelerator radiation delivery system (RDS) for radiation treatment. The MRIS is a vertically gapped 0.35 T horizontal field MR scanner, designed to allow operation with the radiotherapy gantry of the MRIdian Linac system.^1^ In clinical practice, MR images are acquired by employing the integrated transmit/receive (TX) scanner body coil as transmitter while a Torso or Head/Neck surface array coil set acts as receiver.^2^ These receiving coil sets consist of two flexible halves, one placed under and one over the scanned body part. A Torso coil half contains six elements, clustered in two groups of three, while a Head/Neck coil half contains a group of three and a group of two coil elements. The individual elements of phased array radiofrequency (RF) coils function as independent receivers, and the acquired clinical image combines signal information from all available elements. In general, signal intensity detected by phased array coils varies with the distance of the scanned object from its surface.^3^ Surrounding the object equidistantly with the flexed surface coils and applying image filtering, such as prescan normalization, reduces signal non‐uniformities. This filtering method acquires low‐resolution (“prescan”) images with the body coil, which are used to adjust (“normalize”) the signal intensity of images obtained from the surface coils,^4^ thus reducing spatially dependent signal intensity variations.

To ensure accurate patient imaging and irradiation, the proper function of the MRIS, RDS, and their components should be assessed regularly using consistent Quality Assurance (QA) procedures. QA procedures for MRI systems are described in reports published by the American Association of Physicists in Medicine (AAPM)^5^ and the Institute of Physics and Engineering in Medicine (IPEM),^6^ based on standards published by the National Electrical Manufacturers Association (NEMA). Signal‐to‐noise ratio (SNR) is a sensitive global image quality parameter, affected by most MR hardware or software faults. According to the NEMA standards,^7^ SNR can be measured by scanning a large spherical phantom at isocenter in the three orthogonal planes. Produced images can be additionally employed for image uniformity assessments^8^ which are particularly important when using phased‐array coils that may exhibit residual signal intensity variations even after the application of correction algorithms. For such coils it is also important to assess the individual coil elements signal characteristics since each element contributes to the overall acquired image. Monitoring these parameters over time and comparing obtained values to expectations allows for determination of normal system function and detection of diminishing image quality.

ViewRay supplies the MRIdian user with equipment for MRIS acceptance testing. Using this protocol, a fluid‐filled 24 cm diameter spherical phantom is centered inside a hollow plexiglass cylindrical holder, supported by two plexiglass rods. The top and bottom receiving coil halves are attached by Velcro straps around the plexiglass tube, an equal distance from the sphere center. A spin‐echo (SE) sequence with NEMA‐recommended parameters and the option of prescan normalization filtering is accessible in the MR‐only mode, and able to acquire noise‐only images along with the standard signal images for SNR assessments. Regions of interest (ROIs) are manually defined using available tools on the MR scanner computer viewer. Provided ROI statistics (mean, standard deviation, minimum and maximum signal intensity) are used to calculate SNR and uniformity of the filtered or unfiltered combined element images. The SNR for individual coil elements is measured by repositioning the phantom at the locations closest to the elements of interest and by selecting the corresponding element groups for signal reception and “save uncombined.” Signal values are retrieved from an ROI placed on the area of maximum signal intensity, as perceived by the user. For pass/fail decisions, test results are compared against manufacturer‐provided thresholds specific to the aforementioned procedure. SNR and uniformity thresholds should be revisited if MRIdian users would employ a different QA protocol.

Although the described procedure is useful for MRIdian acceptance testing, its implementation for a regular MRIS QA presents several practical issues. Secure positioning of the spherical phantom in the tube is challenging, as the sphere can easily roll, even after using custom fastening materials. If the phantom is not well centered at isocenter on the localizer images, the user needs to repeat the positioning process. Sphere movement during scanning may cause motion artifacts or a decreased phantom cross‐section on acquired images. Phantom repositioning for individual coil element evaluation requires rearranging the phantom and the top coil half, at the cost of additional setup time. Acquiring data at different positions may introduce setup reproducibility errors and signal intensity variations. Moreover, the single slice acquired at isocenter covers only a 10 mm thick volume for each imaging plane, representing a small fraction of clinical coverage. Treatment planning scans expand up to 500 mm to cover the whole head and neck, thorax, abdomen or pelvis of a patient. In addition, manual ROI selection is tedious, time‐consuming and subjective, especially for defining maximum and minimum signal regions required for uniformity and individual element SNR calculations. Replacing manual methods with automated ones would expedite measurements and prevent reproducibility errors.

Alternative commercial solutions that assess multiple MR image quality parameters include the large ACR phantom and MagPhan. The ACR phantom can be placed inside the positioning tube in equal distance from the receiving coils, but features only one slice useful for NEMA‐based SNR and uniformity calculations.^8^ The ACR guidance,^9^ however, does not include SNR measurements and suggests manual ROI definition for uniformity assessments. We found the ACR phantom impractical for monthly QA mainly because the ACR recommended T1 and T2 weighted sequences provide low signal at 0.35 T, requiring multiple averages and long acquisition time (>2 h) to meet the ACR criteria. MagPhan measures image quality parameters including SNR and uniformity at multiple locations albeit with proprietary analysis software^10^ and its shape does not allow for equidistant coil placement and equitable coil element testing. Our site employs the MagPhan weekly using clinical sequences in the treatment mode, mainly for its advantage of 3D distortion evaluation.

Nonetheless, we aspired to implement a dedicated, more sensitive receive coil QA procedure offering large scanning coverage and spatially related assessments of SNR and uniformity with minimal divergence from the original NEMA recommendations and the manufacturer‐provided equipment. We set out to evaluate the function of prescan normalization and study its effects on SNR and uniformity, as this filter is applied on clinical imaging sequences. We aimed to reduce setup time, phantom positioning inaccuracies and motion artifacts by using a stable homogenous phantom centered inside the coils. For coil performance evaluation, we aimed to develop a widely applicable automated open‐source analysis method that eliminates observer bias and defines pass/fail criteria. In this work we present the details and results of the procedure we have developed and implemented for monthly and annual coil QA at our institution for the last 3.5 years and discuss our observations. The presented graphs were produced by custom‐developed code, which is publicly available along with sample data.

## 2 METHODS

### 2.1 Imaging setup

We propose to maintain the manufacturer provided plexiglass positioning tube which guarantees equal distance of the coils from the tube center, but to repurpose the insertable rods and to replace the spherical phantom with a large cylindrical phantom for increased stability and coverage. In our case, we use a commercially available (Siemens Heatlhineers, Erlangen, Germany) 5300 mL cylindrical plastic bottle with curved top (total length 43 cm, diameter 13.5 cm). The phantom is filled with a solution of 3.75 g NiSO_4_ * 6 H_2_O + 5 g NaCl per 1000 g distilled H_2_O, and its center along its length and width was marked on its surface to assist positioning.

**FIGURE 1:**
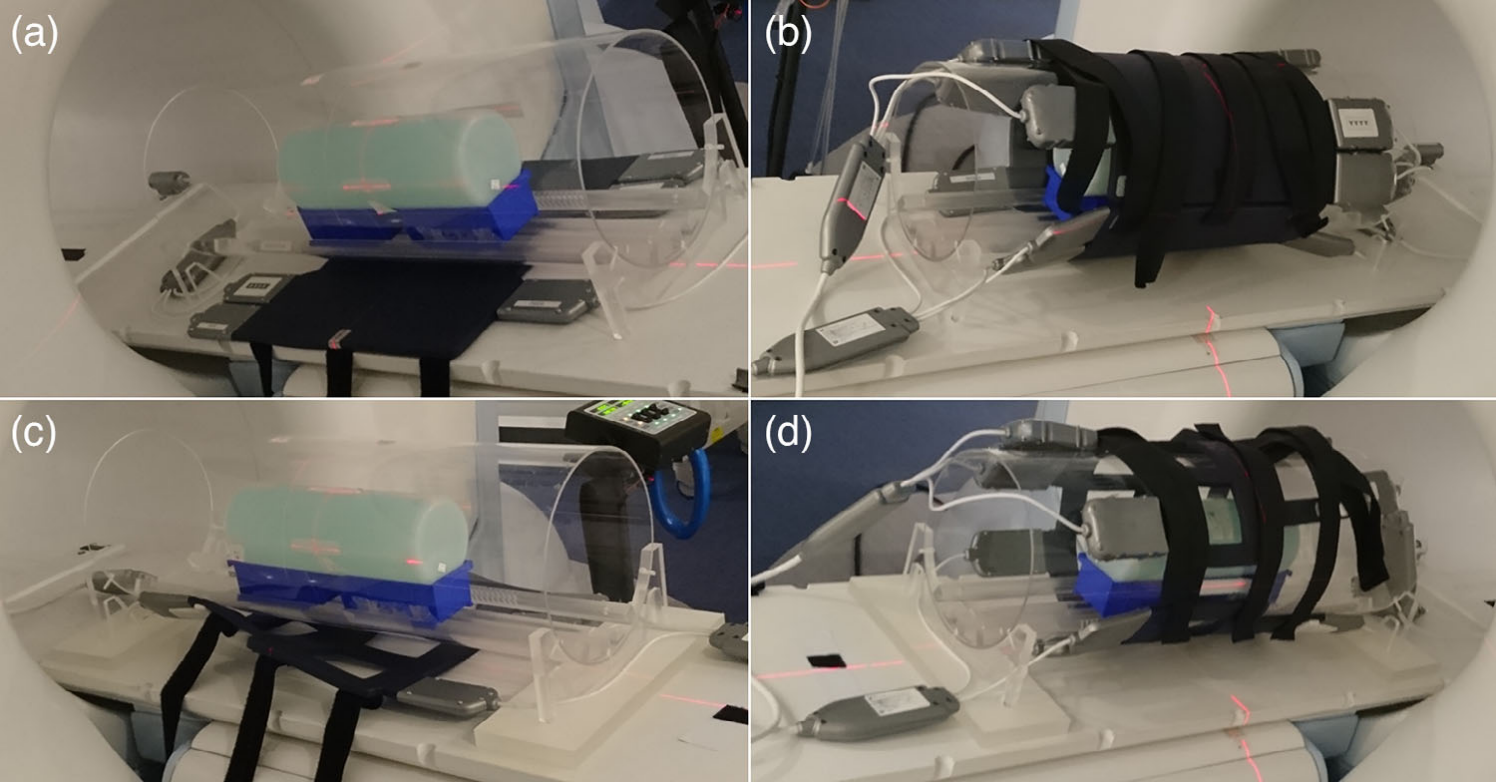
Proposed setup for MRIdian receive coil QA. (a) A cylindrical phantom is placed on stackable bins inside the plexiglass positioning tube above a bottom Torso coil half. Both the tube and phantom are centered and aligned with the external lasers. (b) A closed Torso coil equidistantly surrounding the cylindrical phantom, with its center cross mark matched to the lasers. (c) Tube and phantom setup for a bottom Head/Neck coil half, featuring two custom plexiglass blocks that elevate the tube at the level of the superior section of the coil. (d) A closed Head/Neck coil centered with the external lasers.

Figure 1 displays the proposed setup for coil QA. For Torso coil assessments, the bottom coil half is placed on the scanner couch with the plexiglass cylindrical holder on top. The available plexiglass rods are positioned inside the tube with their thinner side facing outwards, to create a track. The cylindrical phantom is placed in two ULINE plastic stackable bins (model S‐12414, dimensions 7 ½ × 4 × 3″) that can slide on the rods inside the holder tube. To center the plexiglass tube and phantom relative to the scanner isocenter, the marked lines of the tube are matched to the laser lines and to the phantom center markings in the corresponding directions. Custom marks on the bottom coil help to additionally center the coil with the lasers (Figure 1a). Subsequently, the top coil half is attached to the bottom coil half by Velcro straps with its cross marking matched to the axial and sagittal lasers (Figure 1b), and the couch is driven to isocenter. The same procedure is followed for Head/Neck coils, with the exception that the holding tube is raised by positioning two custom 2.5 cm thick plexiglass blocks under its feet in order to avoid overstretching the curved superior section of these coils (Figure 1c,d). As a primary and a spare backup coil set are available for each receiver type, serial numbers (SN) of the top and bottom coil halves are noted and coil placement is kept the same for consistency between repeated measurements. Image QA assessments of the scanner‐integrated body coil can also be performed by employing the same tube and phantom positioning. It is possible to keep any of the two surface coil types connected and unselect them during image acquisition.

### 2.2 Data acquisition

**FIGURE 2:**
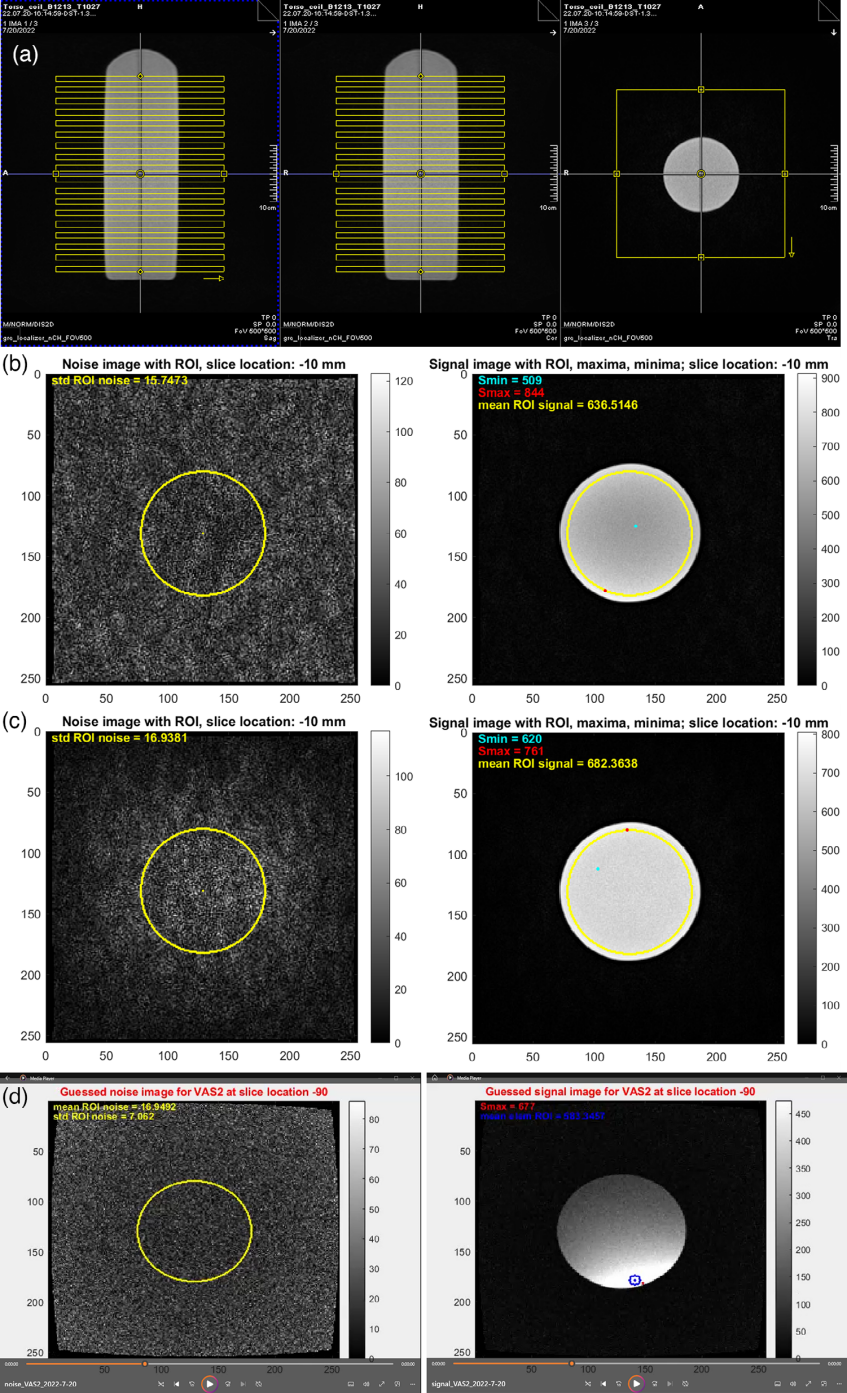
(a) Scanner screenshot showing the placement of axial spin‐echo slices (yellow) on the cylindrical phantom for a torso coil. (b) Unfiltered and (c) prescan normalized Noise and Signal images for a slice acquired near isocenter using the combined elements of this Torso coil. The yellow circle indicates the selected ROI, while the cyan and red dots indicate the minimum and maximum values inside the ROI on the Signal images. The standard deviation, mean signal and extreme signal values obtained from the ROIs for SNR and uniformity calculations are displayed on the images. (d) Produced videos showing the attributed Noise and Signal image for an example element of this Torso coil at an example slice location. The noise ROI and its statistics are shown in yellow. The red dot indicates the maximum value inside the Signal image and the blue ROI marks the area from which the mean signal intensity for SNR calculations was obtained.

Receive coil SNR and uniformity evaluation is performed in MR‐only mode, using a spin‐echo sequence with NEMA recommended parameters (repetition time (TR) 1500 ms, echo time (TE) 15 ms, slice thickness 10 mm, Field‐of‐View (FOV) 300 × 300 mm^2^, matrix 256 × 256, bandwidth 130 Hz/px, flip angle 90°). The sequence includes two measurements: one employs the RF transmitter and receiver as usual for phantom imaging, whereas the transmitter is turned off during the other measurement to receive noise only. Instead of acquiring a single slice in all three orthogonal planes as prescribed for a spherical phantom, we acquire 18 axial slices covering the length of the cylindrical phantom with 100% gap between them (Figure 2a). The FOV is centered at isocenter by default.

For combined coil element evaluation, the sequence is repeated with and without prescan normalization filter using all four coil groups (VAP, VAS, VPP, VPS) of an array coil, and coil combine mode “adaptive combine”, which is the default option in clinical scans. A Signal and a Noise image are obtained from the two measurements of each acquired slice; thus, 36 images are produced for each filtering condition. When the scanner body coil is evaluated as receiver, prescan normalization is not applicable, and only unfiltered images are produced. To assess the individual coil elements of the receive‐only array coils, the spin‐echo sequence is acquired again, selecting “save uncombined” and coil combine mode “sum of squares”. Two data series are produced: the first contains a Signal and a Noise image for each slice and coil element, amounting to a total of 18*12*2 = 432 images; the second contains 36 images, corresponding to Signal and Noise of combined elements for each slice under combine mode “sum of squares”.

### 2.3 Data analysis

The data analysis software was written in MATLAB R2019b (The MathWorks Inc, Natick, Massachusetts, USA) with Image Processing Toolbox and executed on a PC with 64‐bit Windows 10 operating system. The source code is provided on the Harvard Dataverse (https://doi.org/10.7910/DVN/THFI8T) along with example data and instructions for use.

#### 2.3.1 Combined coil element images

Our custom MATLAB code reads the DICOM images of a selected directory, extracts their relevant header information and verifies that one pair of images is available for each slice location. A user interface allows for selection of the coil type (Torso, Head/Neck or Body) and of an overarching directory for saving the QA results of a particular coil set collectively over time.

The Noise and Signal images of a slice are assigned by comparing their maximum signal intensity values, since the Signal image has overall higher intensity in general. The phantom borders on each Signal image are defined by thresholding the image using functions from the OSAQA toolbox by Sun et al.^11^ The phantom center is then specified as the middle of the borders in horizontal and vertical direction of the Signal image. A circular ROI centered at the phantom center with diameter 90% of the phantom width is defined on the Signal image and additionally applied on its paired Noise image. The mean of pixel intensity values inside the Signal image ROI and the standard deviation of pixel intensity inside the Noise image ROI are extracted and used to calculate SNR for each slice according to NEMA standards.^7^ The maximum and minimum signal intensity S_max_ and S_min_ of all the pixels inside the Signal image ROI are used to compute Percent Image Uniformity (PIU) per slice as suggested by NEMA.^8^

This procedure is performed for the filtered and unfiltered combined element data series, as well as for the combined element series with coil combine mode “sum of squares” that is created as a by‐product of the individual elements sequence. An example Noise and Signal combined element image for each filtering condition with applied ROIs and obtained metrics is shown in Figure 2b,c.

To determine if the current SNR and uniformity results of a filtered or unfiltered series pass or fail for each slice location, they can be plotted and compared to a baseline formed by using all previous acceptable results for the corresponding series and the same coil set. A warning or fail indication is displayed if the current results diverge more than 2 or 3 standard deviations from the baseline mean, respectively. Alternative warning/fail criteria of 5%/10% deviation from the mean value can be selected, especially in the case of a short baseline (<10 datapoints). Spare or newly acquired coils can be compared against standard or older coils of the same type in terms of SNR and uniformity using minimum past values of the older coils as reference.

#### 2.3.2 Individual coil element images

The script assessing the individual coil element series for a particular coil set retrieves coil element name and slice location from the DICOM headers and classifies Noise and Signal images of each coil element and slice by comparing their maximum signal intensity. Alternatively, the known acquisition order of the Noise and Signal images can be used for their classification. The circular ROI which was defined on the by‐product combined element series with coil combine mode “sum of squares” is applied on the Noise images of the corresponding slice in the individual element series. The standard deviation of pixel intensity inside it serves as a measure of noise (*std _ROI Noise_*) for each coil element and slice location (example Figure 2d).

As the point of maximum signal intensity on the Signal images may be situated too close to the phantom edge to form an ROI for signal values extraction, these images are smoothed using an averaging filter of size 10 pixels. The point of maximum intensity inside the phantom is found on the smoothed Signal images. A ROI of 5 pixels (5.86 mm) radius is built around the corresponding point on the original Signal images (Figure 2d), and the mean of signal intensity values inside the ROI (*mean _ROI Signal_*) is calculated. The SNR of individual coil elements per slice location is computed according to NEMA.^7^ The Signal and Noise images with their ROIs and extracted values for each coil element are saved as a video running over slice locations. These videos are aimed to document and enable review of selected ROIs and reveal coil element position. Plots of signal, noise and SNR over all slice locations are produced for each coil element.

In order to provide a comprehensive SNR estimate per coil element for longitudinal comparisons, three slices at locations where maximum SNR is observed (110, 130 and 150 mm, + or—depending on coil group) are selected and their mean SNR and its standard deviation are calculated. Pass/fail decisions for a new measurement are made by comparing the current peak SNR to a baseline formed by using all previous acceptable peak SNR values per coil element. A warning or fail indication is displayed if the current results diverge more than 2 or 3 standard deviations from the baseline mean. Alternative warning and fail criteria of 5% and 10% deviation from the mean value, respectively, can be employed for short baselines (<10 datapoints). The individual element peak SNR of different coils can also be plotted and compared.

## 3 RESULTS

### 3.1 Setup

The proposed method was introduced in December 2019, then refined and implemented as a monthly and annual QA from February 2020 till the time of writing (July 2023). The total time to set up the coil QA equipment is 15.5 min on average. The proposed cylindrical phantom lies steady into the employed bins and is well centered at isocenter (Figure 2a), requiring no later repositioning. As all sequences are acquired at isocenter, no user positioning of the FOV is needed. Total acquisition time is 25 min 13 s or 26 min 10s for MRIdian software version V2 or A3i (excluding localizer). The developed MATLAB code is applicable to data obtained under either software version, from any coil type. While the results presented here relate to multi‐slice acquisitions of the cylindrical phantom, our software has also been successfully applied on single‐slice data from the manufacturer‐provided spherical phantom.

### 3.2 Combined coil element images

**FIGURE 3:**
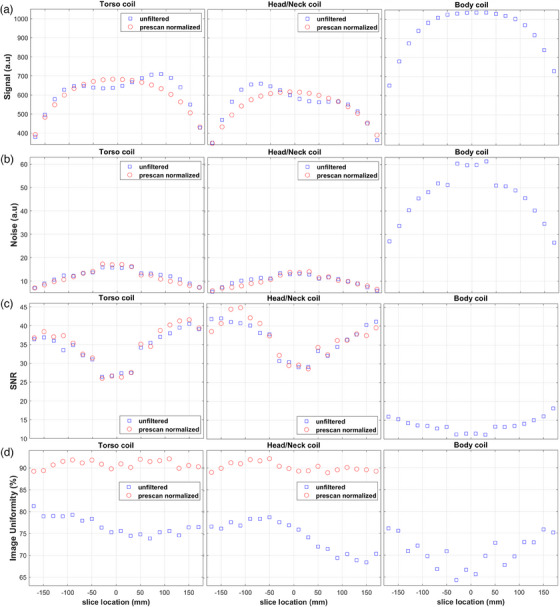
(a) Signal, (b) noise, (c) SNR, and (d) percent image uniformity over all slice locations imaged using the combined elements of a Torso coil (left), a Head/Neck coil (middle), and a scanner body coil (right). All plots share the same X axis, and plots of the same variable have the same Y axis scale. The two receive‐only coils were employed with (red circles) and without (blue squares) prescan normalization filtering, which is not applicable for the body coil.

Executing the MATLAB script calculating SNR and uniformity of combined element images takes around 90 seconds on a 64‐bit Windows PC, including time to manually select directories. Figure 3 displays example results per slice location obtained for a Torso and a Head/Neck coil with and without prescan normalization, as well as for the scanner body coil. It is evident that for unfiltered images, the maximum signal intensity (∼650‐700a.u.) is observed around +90 mm for the torso, but ‐90 mm for the head/neck coil. After prescan normalization, the signal intensity curve for the receive‐only coils shows a similar shape as for the integrated body coil, increasing near isocenter and tapering off further from it. Peak signal intensity is highest for the body (∼1050 a.u.), and lowest for the Head/Neck coil (∼650 a.u.). However, the body coil presents about 5 times more noise, leading to about 3 times lower SNR than the array coils. Owing to its slightly lower noise, the Head/Neck coil ultimately provides similar SNR to the Torso coil, reaching values of 40−45. For all coils, noise peaks at the four central slices of the phantom (locations ‐30 mm to +30 mm), then decreases with distance from the scanner isocenter. Thus, all coils show minimum SNR (∼25‐32) for slices inside ± 30 mm, and maximum SNR (∼40‐45) around ± 130 mm from isocenter. The uniformity of unfiltered Head/Neck coil images is evidently reduced for positive slice locations which correspond to the superior coil section that contains one element less. Uniformity increases from ∼ 75% to ∼ 90% and becomes less dependent on slice location for both array coils after filtering. The body coil presents lower uniformity (∼70%) with higher variability.

### 3.3 Individual coil element images

**FIGURE 4:**
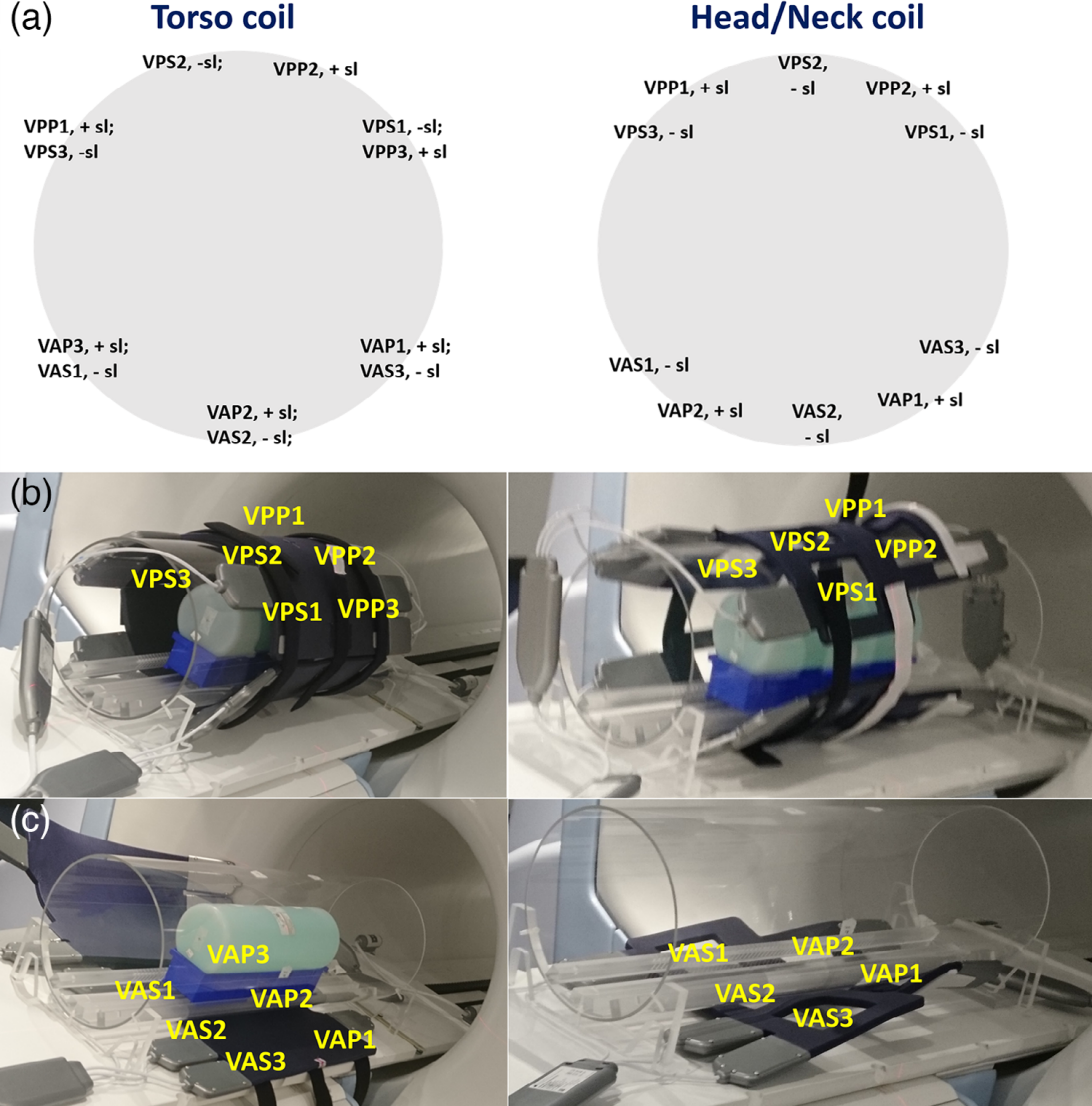
(a) The placement of each coil element name on the circle indicates the approximate location of the area of maximum signal intensity on the axial Signal images of that individual element for the Torso (left) and Head/Neck coil (right). +sl or ‐sl denotes a positive or negative slice location, respectively. The derived coil element position for the top (b) and bottom (c) half of the Torso (left) and Head/Neck coil (right) is marked in yellow. The Head/Neck coil set has no VAP3 and VPP3 elements.

The code analyzing the 432 uncombined element images runs for about 5 min, during which each image with its ROIs is briefly displayed for user verification before been written to a video. By considering the position of the area of maximum signal intensity on these images and the corresponding slice location we were able to derive the physical position of each individual coil element in the phased array. Figure 4a shows a schematic representation of the positions of individual coil elements on the circular cross‐section of the phantom for the Torso and Head/Neck coils, noting a positive (superior) or negative (inferior) slice location. Although three acquisition channels are defined for each element group, the elements VAP3 and VPP3 are physically non‐existent for the Head/Neck coil set. The actual location of each element on the top and bottom half of a Torso or Head/Neck coil is shown in Figure 4b,c, respectively. Note that the anterior position of VP elements and posterior position of VA elements are specific to our workflow and would be reversed if the top and bottom coil halves were exchanged. The third letter “P” or “S” in a coil element name relates to superior or inferior coil element position, respectively.

**FIGURE 5:**
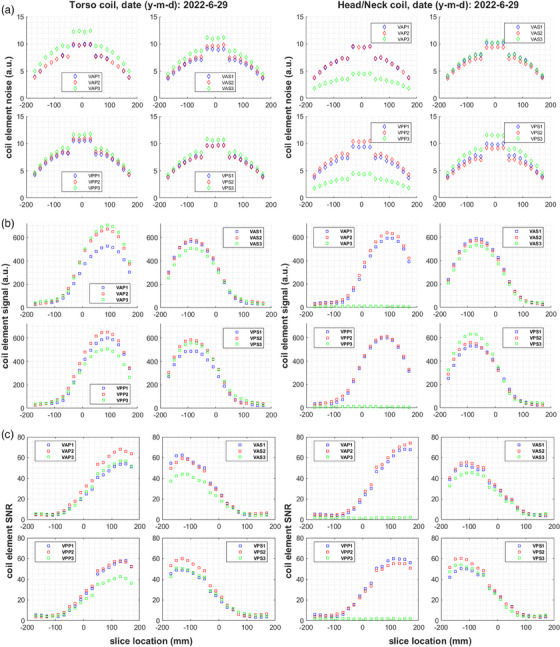
Example individual coil element noise (a), signal (b) and SNR (c) over all assessed slice locations for a well‐functioning example Torso (left) and Head/Neck coil set (right). The 3 elements of each coil group (VAP, VAS, VPP and VPS) are plotted together. Plots of the same parameter have the same axis range.

Figure 5 displays example plots of individual coil element noise, signal and resulting SNR over all slice locations for a torso and a head/neck coil set. In agreement with observations from combined elements images, the four centermost slices show a persistently higher noise (∼10 a.u.), as shown in Figure 5a. Outside this slice range, noise decreases uniformly with distance from isocenter. The recorded noise for the non‐existing Head/Neck coil elements VAP3 and VPP3 is <5 a.u., with a similar spatial pattern. Maximum signal intensity values (500‐650 a.u.) appear around +90 mm for elements of the superior VAP and VPP groups, and around ‐90 mm for elements of the inferior VAS and VPS groups (Figure 5b). Signal images from the VAP3 and VPP3 channels of a Head/Neck coil present maximum intensity <11 a.u. As a combination of the signal and noise curves, SNR reaches its highest value at a different location than the maximum signal: around +130 mm for the superior VAP and VPP, and around ‐130 mm for the inferior VAS and VPS coil groups (Figure 5c). Peak SNR varies slightly for different elements of the same group. The VAP group shows the highest SNR (60‐75) for both coil types. SNR is <3 for the VAP3 and VPP3 channels of a Head/Neck coil where no receiving elements are connected.

### 3.4 Longitudinal assessments for combined and individual element images

**FIGURE 6:**
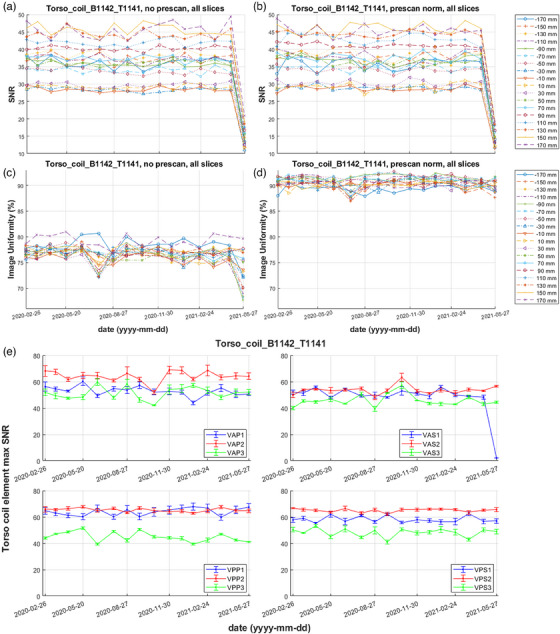
Longitudinal SNR (a, b) and uniformity (c, d) of unfiltered and prescan normalized combined element images of a Torso coil consisting of the bottom half SN1142 and top half SN1141. Each of the 18 scanned slice locations is represented by a different color and marker combination. SNR dropped markedly for the last date (2021‐05‐27). (e) Mean and standard deviation (error bar) of SNR from the 3 slices presenting maximum SNR for the individual elements, plotted separately for each group of this Torso coil set. The maximum SNR for coil element VAS1 dropped to almost zero during the last measurement on 2021‐05‐27.

An example of SNR and uniformity measurements from unfiltered and prescan normalized combined element images over time is shown on Figure 6a‐d for a specific torso coil combination. It is evident that for both filtering conditions SNR is dependent, while uniformity is rather independent of slice location. The last displayed measurement (performed on 2021‐05‐27) presented SNR of unfiltered and filtered images lower than 3 standard deviations from the earlier baseline mean, failing the QA criteria for all slices. Uniformity failed for the inferior (negative) slices of the unfiltered series only. The corresponding longitudinal analysis of “save uncombined” images (Figure 6e) shows the mean and standard deviation of individual element SNR derived from the slice locations which present maximum SNR values: 110, 130 and 150 mm for the superior coil groups VAP and VPP, and −110, −130, −150 mm for the inferior coil groups VAS and VPS. The peak SNR baseline of individual elements suggests that element 3 of groups VPP, VPS, and tentatively VAS demonstrates lower peak SNR than elements 1 and 2 of the same group. On the last displayed date (2021‐05‐27) the VAS1 element failed its SNR QA, while all other coil elements were within tolerance. The detected coil element malfunction explained the SNR reduction on the combined element images. The inferior location of VAS1 (see Figure 4) agreed with the uniformity drop observed for inferior unfiltered combined element images. Knowledge that the VAS group is located posteriorly led to replacement of the bottom coil SN1142.

**FIGURE 7:**
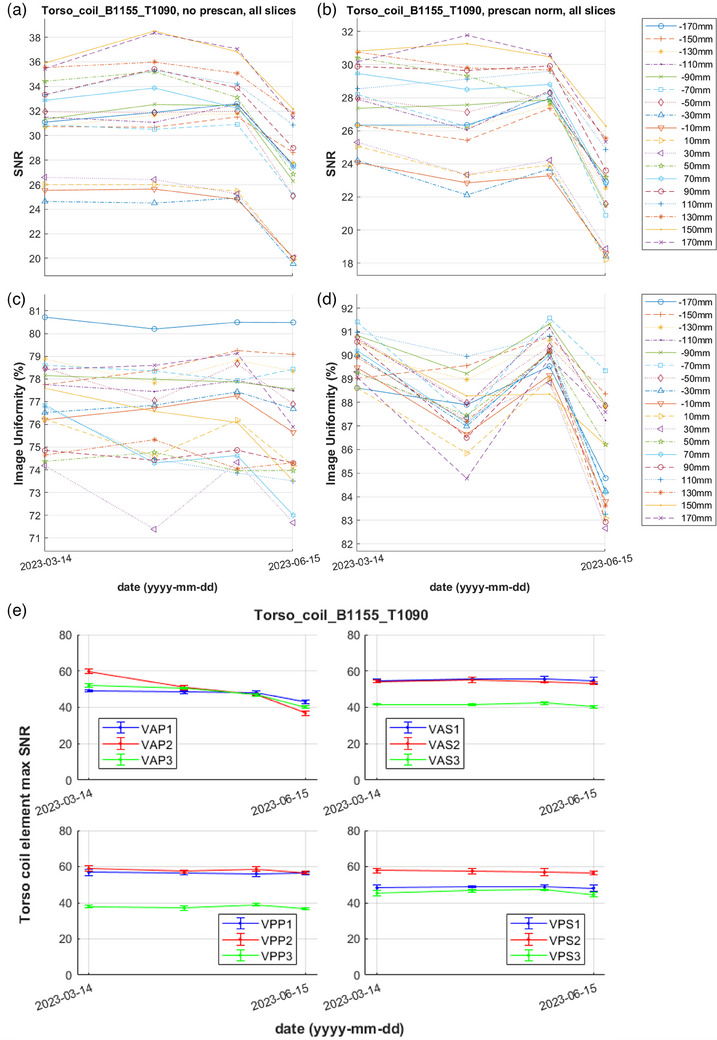
SNR (a, b) and uniformity (c, d) over time for the unfiltered and filtered combined element images of a Torso coil with bottom half SN1155 and top half SN1090. The different lines correspond to the different slice locations. (e) Mean and standard deviation (error bar) of peak SNR from the individual element images for each coil group over time.

Figure 7 demonstrates an example of a subtler coil error caught by the proposed QA method. On 2023‐06‐15 the Torso coil set with bottom half SN1150 and top half SN1090 yielded SNR less than 3 standard deviations from baseline for all prescan normalized slices and for 16 out of 18 unfiltered slices of the combined element series. Uniformity passed for unfiltered images, but a warning was issued for filtered images acquired close to isocenter. Individual element peak SNR comparisons to the baseline revealed that elements of the posteriorly located VAP group failed using either the standard deviation or percentage criteria. Although the SNR decrease was not drastic, after these observations the spare bottom Torso coil half was used clinically instead of SN1150 out of precaution.

**FIGURE 8:**
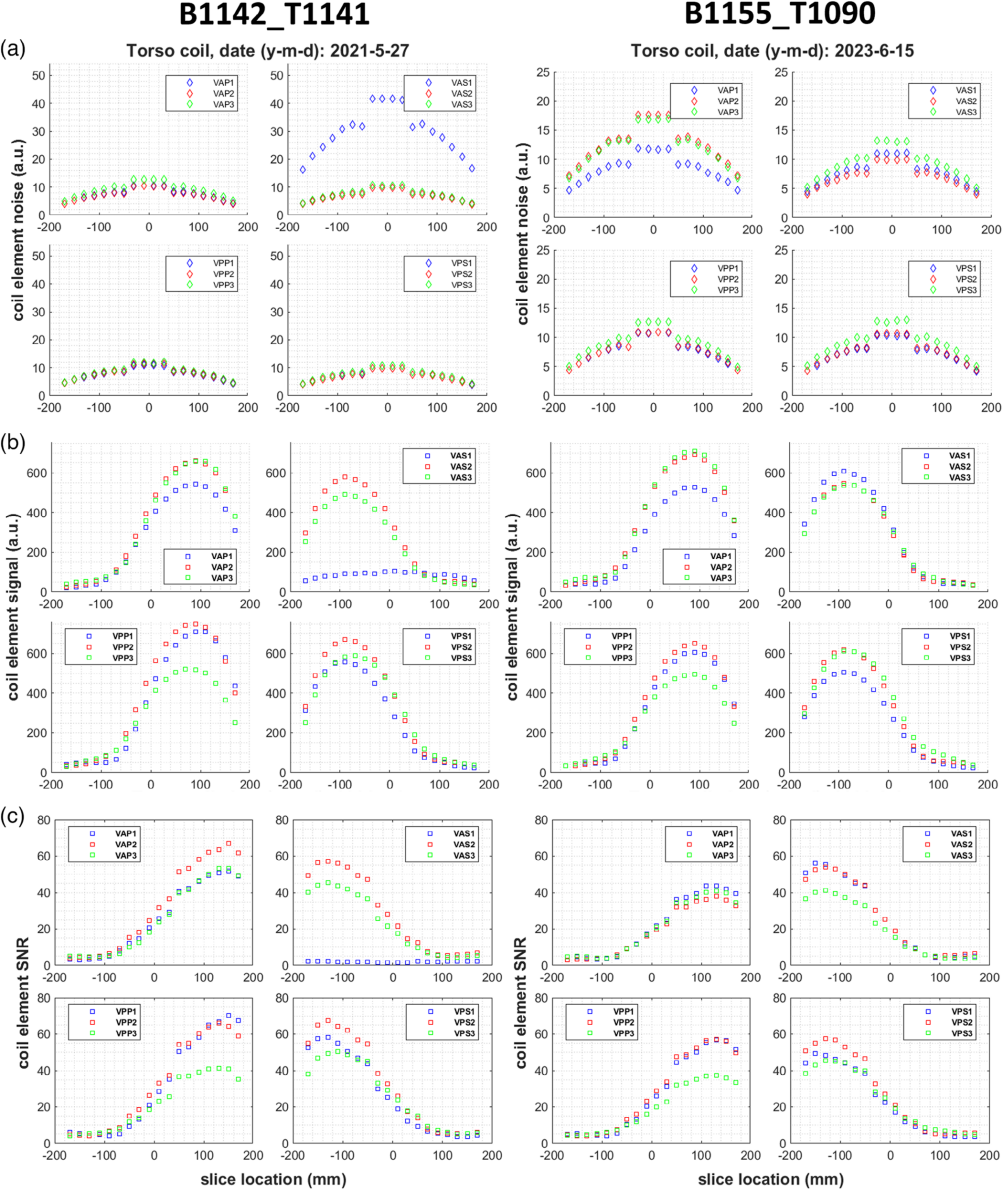
Individual coil element noise (a), signal (b) and SNR (c) over all assessed slice locations for two cases of coil malfunctions. Left: On 2021‐5‐27 the Torso coil with bottom half SN1142 and top half SN1141 presented markedly increased noise and flat signal response for element VAS1, leading to almost zero SNR. Right: The Torso coil with bottom and top half SN1155 and SN1090, respectively, showed slightly elevated noise levels for VAP2 and VAP3 on 2023‐6‐15 causing slightly reduced SNR.

Figure 8 displays the noise, signal and resulting SNR of individual elements over slices for the aforementioned coil malfunction examples. Element VAS1 of Torso coil B1142_T1141 presented three times higher noise than usual on 2021‐05‐27, and an almost flat signal response over slice locations, resulting to an average SNR of 2.1. While individual element signal of Torso coil B1155_T1090 was not affected on 2023‐06‐15, noise was slightly elevated for the VAP2 and VAP3 elements, causing ∼20% SNR reduction for them. The detailed separate noise, signal and SNR plots provided insights into coil faults and explained the findings from combined element images. They helped identify whether drops in SNR were due to increased noise, reduced signal, or a combination of these possible causes.

**FIGURE 9:**
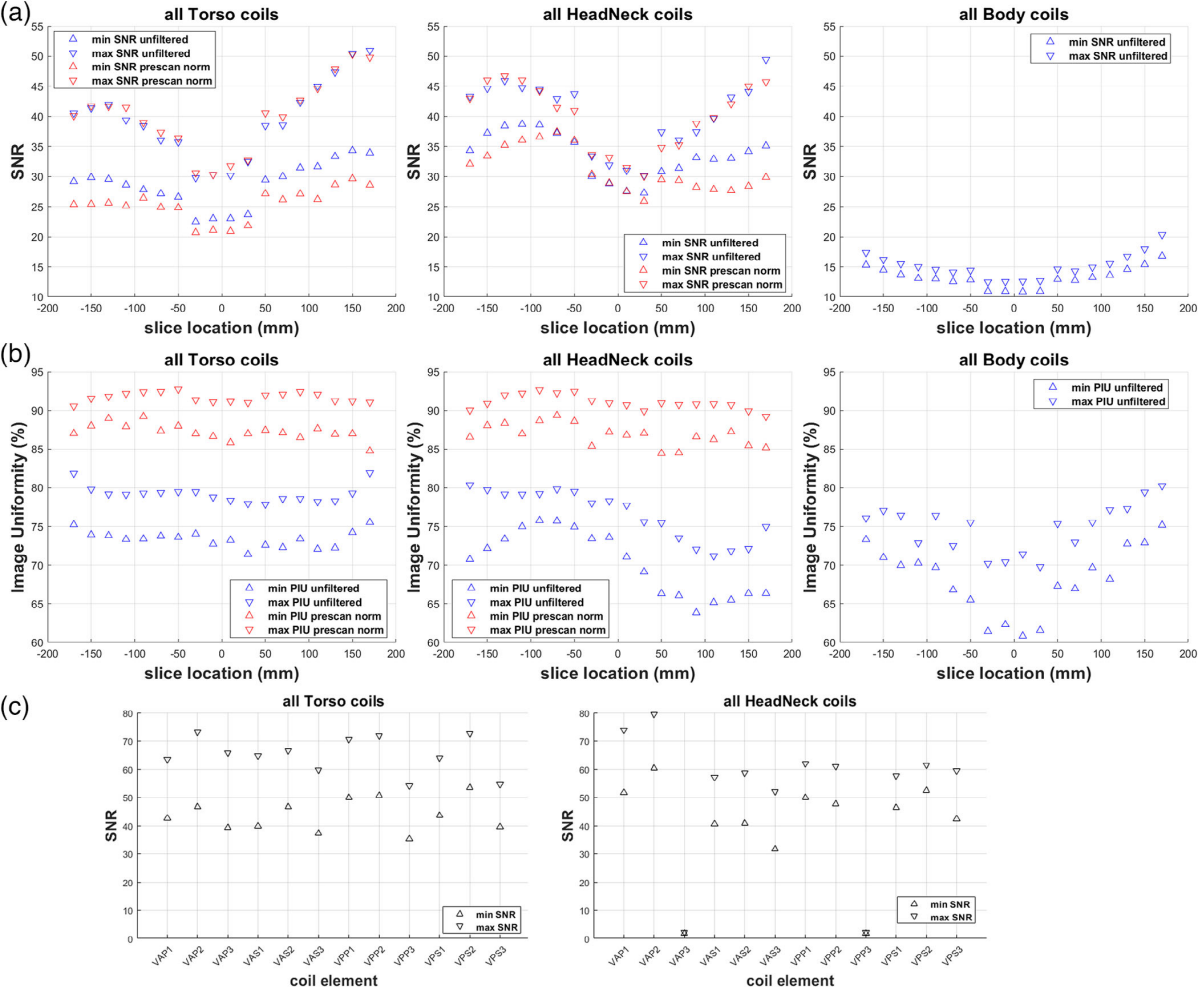
Minimum and maximum SNR (a) and uniformity (b) of all available filtered (red) and unfiltered (blue) measurements from different well‐functioning Torso, Head/Neck and Body coils. (c) Minimum and maximum peak SNR per coil element from “save uncombined” measurements of all acceptable well‐functioning Torso and Head/Neck coils.

During the 3.5 years that the described QA method has been applied at our institution, five cases of decreased SNR and one case of decreased image uniformity due to coil flare were detected for the constantly used Torso coils. In all these cases the individual coil elements causing the issue were identified, and findings were communicated to the service engineer. Malfunctioning Torso coil halves were replaced by the manufacturer. No SNR or uniformity issues were observed for the seldom employed Head/Neck coils. The function of spare or newly introduced coil halves was tested by plotting and comparing their image quality parameters against earlier data of properly functioning coils of the same type, keeping in mind the possibility of inter‐coil variations. Figure 9 illustrates the range of SNR (a) and uniformity (b) values per slice location from all available coils of each type and for both filtering conditions. It is evident that, despite spatial variability, SNR was always >20 for well‐functioning receive‐only coils, and > 10 for the Body coil. Prescan normalization did not significantly alter maximum SNR, but reduced minimum SNR for both receive‐only coil types. Image uniformity was always >70% for the Torso and >60% for Head/Neck and Body coil without filtering. After prescan normalization, image uniformity lay in a similar range (>80%) for the receive‐only coil types. Figure 9c plots the lowest and highest mean peak SNR (decreased or increased by its standard deviation, respectively) per coil element from all acceptable Torso and Head/Neck coil measurements. Elements 1 and 2 of all coil groups exhibited SNR ≥ 40, while SNR of existing elements 3 was > 30.

## 4 DISCUSSION

The proposed QA setup using a cylindrical phantom supported by plastic bins proved stable, contrary to the spherical phantom. The setup was reproducible by aligning the bins with the plexiglass tube, and the tube and phantom with the external lasers. The phantom was well centered and no repositioning was necessary. Scanning at isocenter prevented user‐specific variations of FOV placement. Acquiring axial slices only was a limitation of our method, yet they spanned a large area of 34 cm (± 17 cm from isocenter) on the Z axis, close to the coverage of clinical torso and head/neck scans, and of 13.5 cm on the X and Y axes. In our case, the test object selection was driven by its on‐site accessibility, but different homogenous phantoms can also be used. The employed spin‐echo pulse sequence parameters followed NEMA recommendations, except for the use of multiple slices. Single slice acquisition avoids interference between adjacent slice profiles (“cross‐talk”) caused by RF‐pulse shape imperfections. Nevertheless, our implementation of 100% slice gap also prevents slice cross‐talk.^12^ Moreover, a spherical phantom would need to be repositioned closer to the coil groups to scan a single slice for individual element analysis. Multi‐slice imaging of a cylindrical phantom saves setup time and eliminates the inter‐scan variability between repeated scans of the same phantom in different positions. The large cylindrical homogenous phantom facilitated detailed assessments of signal, noise, SNR and uniformity with distance from isocenter which have not been performed in other MRIdian QA studies,^13^, ^14^ which employed commercial test objects.

The automated analysis offers a fast, objective and consistent data handling method. The developed MATLAB scripts work for multi‐slice and single slice acquisition, are independent of phantom or acquisition orientation, and are applicable to data obtained with all MRIdian coils and both V2 and A3i software versions. Instructions in https://doi.org/10.7910/DVN/THFI8T describe the workflow and the recommended folder structure. All analysis steps can be verified by the user since images, ROIs, plots, and quantitative results are displayed and saved. The open‐source code can be adapted to accommodate different test objects and MR systems. The SNR calculation algorithm can be applied to Noise and Signal images, and the uniformity calculation algorithm to Signal images of any homogenous phantom. Phantom borders are automatically detected using image thresholding, and ROI size is adjustable to the test object size. The diameter of our combined element analysis ROI was defined as a high percentage (90%) of the phantom width because the circular cross‐section of our test object (13.5 cm) is rather small. The small phantom diameter and consequently ROI size represents a limitation of our study. Future use of a larger cylindrical phantom would improve the presented method, though such a phantom may be heavier and more difficult to handle. SNR is expected to be higher and uniformity lower for bigger ROIs defined on larger phantoms, since signal intensity would increase closer to the surface coils surrounding the test object.^3^ For individual coil element SNR calculations, forming a ROI around the maximum signal intensity point of the smoothed instead of the original Signal images ensured that the entire ROI is situated inside the phantom. The ROI size was selected proportionally to the cross‐section of our test object and can be increased for larger phantoms.

Our SNR and uniformity assessments of unfiltered and prescan normalized combined element images evaluated the impact of prescan normalization on these image quality parameters. Our observations that it smoothed spatially the signal response of the receive‐only coils in a manner similar to the body coil confirm the theory about the function of this filtering method. Its application increased image uniformity significantly but reduced slightly the minimum SNR for both receive‐only coil types. Acquiring unfiltered data for SNR and uniformity QA is concurrent with NEMA recommendations,^7^, ^8^ yet including prescan normalized data makes sense as this filter is applied in clinically employed sequences. For future coil QA under time pressure, it may be sufficient to collect and analyze filtered combined element data only, bearing SNR reduction in mind.

By evaluating mean signal intensity at different slice locations, we were able to determine areas of higher and lower signal for each surface coil type, which coincided between unfiltered combined and uncombined element images. The identified peak signal location of ± 90 mm agrees with the manufacturer provided information that coil element groups are located at ± 100 mm distance from the center of a flat surface coil. Our acquisition skips the exact positions of ± 100 mm axial distance from isocenter due to the employed 100% slice gap. The four middle slices, covering ± 30 mm around isocenter on the Z axis, present consistently higher noise for all coils, for both combined and uncombined element images. Noise decreases with isocenter distance outside this range. Presumably, the increased noise around isocenter is caused by the split magnet design which leaves a 28 cm gap between the two magnet halves for the gantry, and the lack of windings and electrical connections across a 20 cm central section of the split gradient coil.^1^ As noise images were acquired without RF excitation, the noise level received by the various coils is expected to be independent of the employed phantom.

Combined element image SNR is minimized at the four central slices as a consequence of the elevated noise. Peak SNR from either combined or uncombined element images is shifted further from the peak signal location to about ± 130 mm away from isocenter because noise is reduced for slices near the FOV edge. Maximum SNR is found superiorly for Torso and inferiorly for Head/Neck combined element images. Overall, obtained SNR values were in a similar range as those reported in^15^ for the ACR phantom and a Torso coil. “Save uncombined” image assessments reveal details about the noise and signal response of individual coil elements, their physical location, and how they affect overall image quality. Mean peak SNR differences between elements of the same group are observed for both coil types, with element 3 presenting the lowest values of the anterior Torso groups VPP and VPS. This SNR differentiation presumably reflects differences in technical characteristics of the individual elements of a coil group. The non‐existing VAP3 and VPP3 elements of Head/Neck coils are identified by their negligible SNR. The superior groups VAP and VPP yield on average higher peak SNR than the inferior groups VAS and VPS for both coil types. This finding corroborates the superior location of peak SNR for Torso coil combined element images. The contrary inferior location of maximum SNR for Head/Neck coils is explained by the smaller number of elements in the superior coil groups VAP and VPP. Taking advantage of the areas offering maximum SNR may be difficult in standard clinical practice where the clinical area of interest is usually placed at isocenter. Nevertheless, pinpointing these areas may benefit research applications requiring the highest possible image quality. For example, we performed nanoparticle studies at our institute by placing the samples closest to the superior groups of a Torso coil set.

To evaluate the function of a particular coil we define passing, warning and failing thresholds for each assessed parameter according to divergence from its earlier baseline. The standard deviation from the baseline mean appears as a more suitable criterion for longer baselines while a percentage of the mean is more reliable for fewer past datapoints. The individual baseline approach arises from the exploratory character of our measurements and acknowledges possible minor variations in performance between different coils and their elements. Past findings for the same coil were used to set fixed thresholds below which SNR and image uniformity were considered to fail. New coils were tested by comparing their SNR and uniformity results to the lowest values observed for well‐functioning coils of the same type. The reported SNR and uniformity minima over all acceptable measurements can serve as reference for testing other MRIdian systems, with the limitation that these thresholds are valid when QA is performed following the described procedure. Minimum SNR and uniformity may vary for different phantoms and imaging protocols. Including clinical sequences in future QA would increase its clinical relevance.

The employed QA workflow detected several instances of malfunction for the constantly used Torso coils, and no issues for the seldom employed Head/Neck coils. Torso coil failures are probably due to wear and tear, as these coils are used for almost all clinical examinations. Comparing information from combined (filtered and unfiltered) and uncombined element series confirmed the reduction of image quality parameters and identified the malfunctioning coil elements thanks to their derived location in the multi‐slice FOV. Our QA method has caught not only cases of dead coil elements obviously worsening image quality but also cases where SNR was slightly impacted by the slow deterioration of coil elements recording more noise and/or less signal than in the past. Comparing image quality parameters for all coils of the same type and evaluating different coil groups separately proved useful when surface coil halves were replaced.

## 5 CONCLUSION

We introduced and implemented a multi‐slice imaging workflow for monthly MRIdian Linac coil QA and developed an open‐source software offering automated, fast and objective data analysis. Our method provided useful insights into coil and image filtering performance, spatial variations of SNR and uniformity, and coil element location for all available receive coil types. The defined SNR and image uniformity criteria identified cases of faulty coil elements and were useful for evaluating new coils.

## AUTHOR CONTRIBUTIONS

Evangelia Kaza and Christopher Williams developed the concept of a dedicated coil QA using a large FOV phantom. Evangelia Kaza designed and applied the proposed QA setup and acquired the data. She developed, tested and implemented the analysis software, interpreted the data and drafted the manuscript. Christopher Williams provided advice on the software development and data analysis. Both authors have revised the work critically, approved the final version and agree to be accountable for all its aspects.

## CONFLICT OF INTEREST STATEMENT

The authors declare no conflicts of interest.
